# Lawson Wilkins and my life: part 2

**DOI:** 10.1186/1687-9856-2014-S1-S3

**Published:** 2014-05-28

**Authors:** Claude J Migeon

**Affiliations:** 1 502 Somerset Rd, Baltimore, MD 21210, USA

## A few days of fun in Baltimore (January 1953)

I arrived in New York City, Kennedy Airport, took a taxi ride to the Pennsylvania train station and I was on my way to Baltimore. I was exhausted, a little sad to leave family, as well as apprehensive about my job in Utah.

The three-hour ride gave me a chance to ask myself if I had made the right decision. I could have stayed in France, and found a position as a pediatrician in the provincial city of Reims, capital of Champagne and two hours from Paris by train. I would have found a wife and settled down with a nice family. I guess Wilkins had more confidence in my ability than I probably had. I had no easy answer. But then a short taxi ride to 501 Edgevale Road and I was at the Wilkins home. Both Lucile and Lawson were very warm. I had brought gifts, a bottle of Remy Martin Cognac for Lawson and a scarf for Lucile. We had a wonderful supper.

The next day, we had breakfast and I accompanied Lawson to Hopkins. I had fun saying hello to all the people I had met before: the secretaries, Alfred Bongiovanni, Walter Eberlein, and George Clayton, the technicians: Mary-Ellen Crafton and Betty Youngblood, Dr. Joseph, and Dr. Helen Harrison. This was like returning home.

During my time at the Wilkins home, I had to visit the garden, a pride of Dr. Wilkins. There was not much to see in January, but Lawson showed me the trees behind the house and he discussed his plans for the spring. We talked at length about some of the patients I had met in the previous two years. Mrs. Mahool, the mother of Lucile, came to visit.

But the time passed quickly and I had promised to go to Salt Lake City as fast as I could. So after five days, I had to go back to New York to take my plane. We were late driving to the station for the train to NYC. Dr. Wilkins had insisted that I should take the Baltimore and Ohio train rather than the Pennsylvania train. Unfortunately there was some accident on the way to the station. Lawson drove on the sidewalk to avoid the problem and we made it on time.

Lawson asked me how much money I had with me. He decided that it was not enough and forced me to take two $50 bills. I promised to keep him posted on my life in Utah. We said good-bye and I was on the train, a little sad and a little scared of what I would find in the City of the Mormons.

## To Salt Lake City

The United Airlines plane I boarded at Kennedy stopped in Denver en route to Salt Lake City On landing in Salt Lake City, the plane just touched the runway but it roared back its motors and we went back into the air. We were told that there was a problem on the runway. We made a short turn and then tried again to land, this time successfully. When I wrote to Dr. Wilkins, I noted that this incident was frightening and I wondered if it was a bad omen.

Salt Lake City is on the floor of prehistoric Lake Bonneville, caught between the Wasatch Mountains. I found it was blessed with a very bright sun and full of pure air – but not much was going on. The airport was 10 minutes from downtown. The streets were amazingly wide and everything was very clean. I explained to Lawson that one has a strange feeling, breathing a little faster, because of the altitude of 1320 meters.

**Figure 1:**
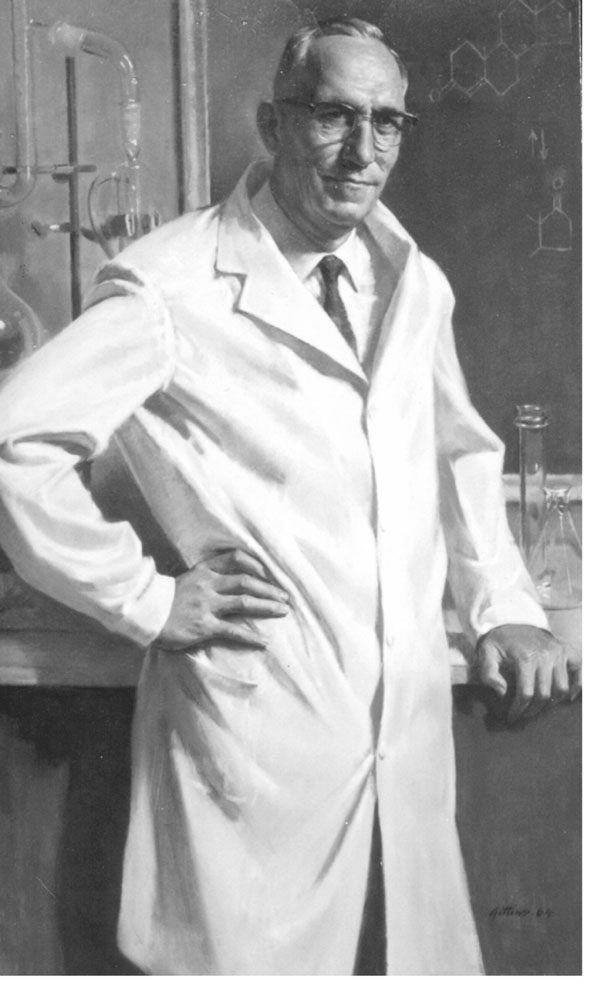
Leo T. Samuels, Director of the Department of Biochemistry at the University of Utah School of Medicine in Salt Lake City.

After a short taxi ride, I was passing by the central buildings of the University of Utah. A turn to the right, and we were in front of an austere construction, the medical school. This building included only the basic sciences. On the second floor was the office of the Department of Biochemistry. Here, I was welcomed warmly by the department secretary, Margerie Riches. Dr. L.T. Samuels (Figure 1) was out of town for a few days, but Margerie, in the most comforting way, took care of making arrangements for a small apartment near the University grounds. She made sure that the place would be affordable. By that time, Dr. John Plager and Robert Martin were in the office. Both offered to drive me to check out my potential apartment. And then we went to see my new home. Everyone was very kind and helpful. I was invited for supper by Hans Reich, a fellow from Switzerland and his wife Elsa.

The next day, I was introduced to my laboratory, a good-sized room with a central bench, and benches on both sides. At the end of the room, there was a large window and a desk. I was also introduced to my three technicians: Anne Keller, Carma Darley, and Patricia Wall. I met a dishwasher, Carl Paul, a good Mormon. Everyone was pleasant and helpful. I explained all this in my letter to Dr. Wilkins. He answered back, stating that he was not surprised by the warm welcome; he knew many people at the University of Utah.

## Physiology and pathology of adrenal function (1953-1955)

I rapidly learned that the Utah group had pioneered the assay of cortisol in human blood: the Nelson-Samuels method. The new tool appeared to have involved most of the clinical departments of the hospital: Drs. CH Hardin Branch and Eugene Bliss in Psychiatry, Drs. Frank Tyler, Avery Sandburg, Harold Brown, Roy Slaunwhite, and JZ Bowers in Medicine, Dr. Vincent Kelly in Pediatrics, Dr. Holmstrom in Gynecology, Drs. John Plager, Kris Eik Nes, and Hans Reich in Biochemistry, and Dr. AB French in Physiology. All this activity appeared to be directed by Dr. Leo Samuels. Dr. Wilkins was informed, as I reported to him that we studied the diurnal variation of cortisol in men and monkeys, the effects of surgical and psychological stress on cortisol secretion, the effects of ACTH under various clinical conditions, the effects of insulin, histamine, and bacterial pyrogens, the response to electroconvulsant therapy and whole body irradiation.

I sent the pre-publication papers of the group to Dr. Wilkins and he gave his comments. In three years, I was an author on 20 scientific papers.

I had fun with the study of psychological stress. The subjects were medical students who were taking their final oral exam in Medicine given by Dr. Maxwell Wintrobe. He had designed the special tube for determination of hematocrit when he was at Hopkins before becoming the Chief of Medicine at Utah. He had the reputation of being very demanding and the students were anxious about his exam. So we took three blood samples 2-3 weeks before the exam and one immediately before and after the exam. Psychologists determined the level of anxiety of each student. The results showed no correlation between the anxiety and the degree of increase of cortisol; so much for psychological stress and cortisol response. But, in most cases, there was some increase of levels following the exam, demonstrating the potency of an oral exam with Dr. Wintrobe.

## My social life at the University of Utah Medical School

When Lawson wrote that he hoped that I did not regret following his advice, I had to tell him that everybody in the Department of Biochemistry was very kind and pleasant. I explained that all the staff, technicians, and secretaries had a very cordial relationship. Everyone worked very hard including Saturdays and parts of Sundays. But on Wednesdays at noon, the laboratory closed down for skiing! I understood that starting early in the fall and through late in spring, this was the thing to do. Wednesday had been chosen because the cost of the lift was one-third of that on weekends, and there were much fewer skiers on the slopes.

**Figure 2:**
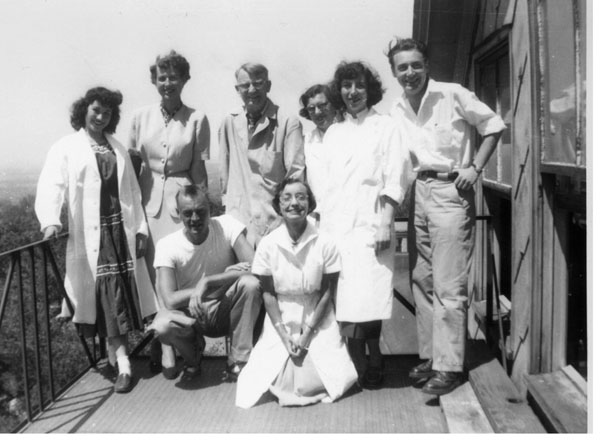
The group of workers in the Department of Biochemistry with Mrs. Samuels in front on a knee (1954).

I was told that I had to learn to ski. Hans Reich gave me a pair of skis. Someone else provided the pants and jacket, and a third, the gloves and poles. On the first Wednesday, at noon, two or three cars would be ready. Everyone had a sandwich to eat en route. (Figure 2)

I was given lessons on “how to fall,” which was apparently very important. Then I was put on a seat of the lift and taken to the top of the slope. From there, I looked at the bottom, but was advised that I could snow plow, going down from one side of the slope to the other. Margery Riches was kind and was there to pick me up when I fell. After several falls, I managed to reach the inn at the bottom, where I was congratulated by my friends.

The beauty of the Wasatch Mountains of Utah is incredible. I explained to Lawson the importance of skiing. Several of the medical staff, like Dr. Cartright, went in spring to evaluate the amount of snow in various locations in order to predict how much snow would melt to furnish water for the irrigation of the cultures of the farmers.

Also, on the 4^th^ of July, young skiers go to the top of mountains surrounding Salt Lake City and ski down at night with a torch in hand: a beautiful spectacle!

While I did learn to ski, I was not very good at it. In the spring of 1954, the group went to Snow Basin. It was a beautiful sunny day, but it was icy on the slopes. I fell and broke my left fibula. Hans Reich, who saw me fall, decided that I was fine, so he went down with the rest of my friends. As it was noon, the lift stopped running, everybody had their sandwiches, while I sat halfway down the slope, unable to move. Eventually, the lift restarted, the ski patrol picked me up and Dr. Samuels’ wife, Barbara, who was part of our group of skiers took me to the emergency room in Salt Lake to obtain a cast. For a few days, I stayed at the Samuels’.

When I was able to move with a walking cast and a cane, I went back to work at the medical school. Young students that I did not know would salute me with “how is your skiing these days?”

The next year, I was quite willing to pass on skiing, but because I was being called a quitter, I went skiing again. I have to admit that I was extremely careful and I never skied again after returning to the East Coast.

After skiing, there was usually a visit to a Mexican restaurant for tacos and enchiladas.

During the summer, the trips to the mountains were for picnics with a fire for cooking hot dogs and marshmallows.

Part of my indoctrination was that I had to attend concerts. The Salt Lake City Orchestra, directed by the renowned Maurice Abravanel, was quite excellent. The Mormon was an impressive group of wonderful singers. Many Mormons competed to become part of the choir. The Requiem of Verdi by the orchestra and the choir was incomparable to anything I had heard before or since. However, one trouble with the concerts was that the acoustics of the hall were rather poor. The oval shape of the hall distorted the sound. In only two parts of the auditorium could you hear the music just once, everywhere else there was an annoying echo.

The University Theater was another function I was told I must attend. The students were good actors. The production of “Julius Cesar” was almost too much to take. Hordes of Roman soldiers surged throughout the theater, dressed with their short skirts, carrying lances, and epees.

The football games between the University of Utah and Brigham Young University were a heated rivalry. Our entire department always was eager to cheer for U of U! I was amazed that some of the people who attended the games drank from metal flasks (probably scotch). As Lawson knew Mormons do not drink any alcohol, on my visit to Baltimore, he suggested I have an extra glass of liquor to catch up for the dry spell.

One time, I was visited by Jim and Sylvia Tait, the British researchers who first isolated aldosterone. I invited them to dinner at the best restaurant in town at the Hotel Utah. I had bought a bottle of good, expensive French wine from the State-run alcohol distribution center, the only place where alcohol could be purchased. I knew that the restaurant would permit wine in glasses, but the bottle had to be kept under the table.

At the appropriate time, I asked the waiter for a corkscrew. He blushed and said he did not have one. He went back to the kitchen and eventually came back, but with no corkscrew. So I explained to the Taits that I would see if I could find one in a drug store. Having no success. I kept the bottle between my legs throughout the dinner. Jim never let me forget this fiasco and his first words, whenever he saw me, were to inquire whether I had kept the habit of keeping a corked bottle of wine under the table each time I went to a restaurant. When I told Dr. Wilkins about this ordeal, he did not find it funny.

## The Ciba Foundation: London (April 1954)

I wrote Dr. Wilkins that I had been invited to speak at the Colloquium on Endocrinology (Vol 8) organized by Dr. Gordon Wolstenholme and Miss Margaret Cameron to be held in London. The Colloquium was entitled “The Human Adrenal Cortex.” I told him that Dr. Frank Taylor was going to present our work on the effects of epinephrine on the metabolism of 17-hydroxycorticosteroids in humans. I added that I was going to present my work on “Adreno-cortical Function and Plasmic 17-ketosteroids in Man.”

Lawson answered that he was also going to that meeting and would give a paper in collaboration with Al Bongiovanni, George Clayton, Mel Grumbach, and Jud Van Wyk on the steroid abnormalities in and treatment of congenital adrenal hyperplasia.

Bongiovanni had shown that their patients had an excessive secretion of 17-hydroxyprogesterone and progesterone in addition to the adrenal androgens.

Lawson and I made plans to see each other in London. I took a plane from Salt Lake to New York and then the TWA plane to London, with stops in Reykjavik, Iceland and Shannon, Ireland.

**Figure 3:**
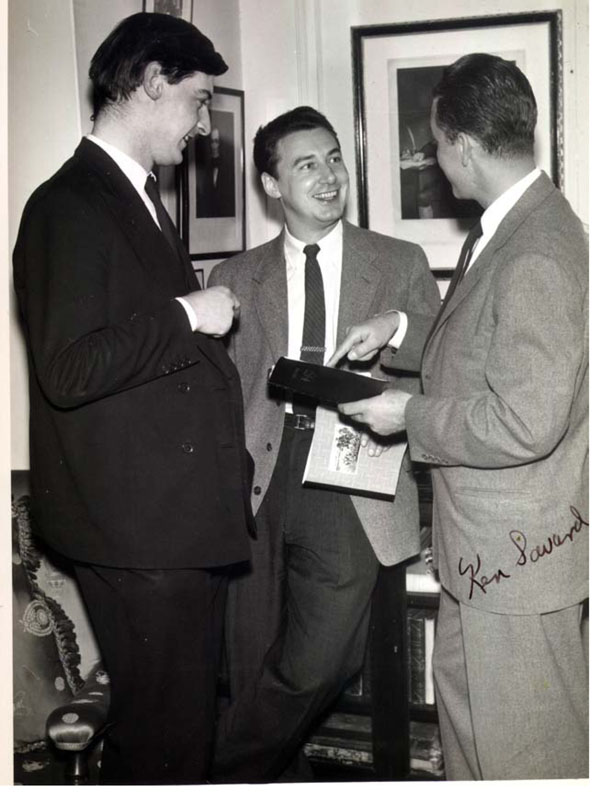
The CIBA Foundation in London. From left: Ian Bush, Claude Migeon, and Kenneth Soward. (April 1954)

The senior investigators, including Lawson, were accommodated at the Ciba Foundation, 41 Portland Place. I had a hotel close to the Foundation, sharing a room with Dr. Ian Bush, a great friend. Drs. Ken Savard (Figure 3) and Claude Giroud of Montreal were next door. Several other young investigators were with us as well.

I had a long conversation with Lawson. He told me that he was very pleased with his present fellows. Bongiovanni had worked with Liebermann in New York and had fascinating data which explained the pathophysiology of CAH. In addition, Grumbach and Van Wyk were working very hard.

There were several special meals at the meeting with toasts “To the Queen,” which Dr. Wilkins loved.

The scientists who contributed were the world experts in the field. First, there were the studies of aldosterone: Wettstein and Neher of Basel, Dr. Venning from Montreal, Sylvia Simpson and James Tait who had just isolated aldosterone, Dr. Gaunt from the US, and Dr. Prader from Zurich.

Then there were the “clinicians” like Lawson, Drs. George Thorn, and Andrea Prader.

Dr. Frank Taylor did a great job in presenting our data and my own paper was well received but with many questions raised about the testicular androgen testosterone.

Peggy Cameron, one of the conference organizers, had visited us in Salt Lake City, and it was fun to see her again. I had a chance to talk to many of the attendees, and it was very instructive.

A bus was taking some of us to the airport. I had to say goodbye very quickly to Lawson before heading back to Salt Lake City and the laboratory with my laboratory assistants.

## An important visit to children’s hospital of Harvard (September 1955)

In 1955, John Crigler, who had been a colleague my first year in Baltimore, was now settling into his new position as the head of the Pediatric Endocrine Clinic at Harvard Children’s Hospital. We had talked about me joining him and establishing a hormone research laboratory. He was very anxious to have me, and invited me to Boston to discuss my needs. I also was asked to give a lecture, which was a little intimidating. As I was walking toward the conference room, Dr. Charles Janeway, the chief of Boston Children’s Hospital and a wonderful person, noticed my nervousness. He told me that he always had to go to the bathroom to empty his bladder and wondered if that was my problem, too. We both went to the men’s room and urinated together. This common humanity relieved my apprehension and my lecture went very well.

My talk was followed by a visit to the Jimmy Fund Cancer Research Building, the domain of Dr. Sidney Farber. Dr. Janeway told me that I had to be introduced to him, and that we would have lunch together.

We went to the top floor of the Building, the place where Dr. Farber had established his library. All the books on the shelves were hiding behind wooden panels, and a table was set for four people. I was introduced to Dr. Farber, whom I knew was an important decider about new faculty at Children’s Hospital.

Then, the maid (cook) brought a plate of chicken to Dr. Janeway, another plate to John Crigler, and one to me. Finally she returned with a huge steak, and presented it to Dr. Farber who explained, “Too bad, I cannot eat chicken.” The memory of his special lunch has remained with me for all these years. When I told Lawson about it, he had a big laugh.

After I had gone through all the steps required of the visit, I took the train back to Baltimore to discuss my plans with Dr. Wilkins. Both John Crigler and Dr. Janeway had given me encouragement. We discussed what I would need for my research and everything seemed to be acceptable.

Because I had not told Wilkins of the major reason for my Boston visit, he was a little shocked when I told him that I was thinking of joining John Crigler. He asked, “Why don’t you come back with me in Baltimore instead.” I answered that I had believed that was not possible. I told Lawson that my research would require a laboratory room with a special spark-proof hood and also a small room with 37ºC constant temperature for paper chromatography using the Bush system. Lawson interrupted, suggesting that I stay a couple of days to discuss the possibility of filling my requirements. The next morning, Lawson, brought me to one of the engineers of the hospital. We first went to the 5^th^ floor of Harriet Lane home to check on space. They identified a small room which could serve as the 37ºC constant temperature room for chromatography. The old lab in the middle of the floor could be equipped with a hood, and its working bench could support cabinets that could be used for glassware. For an office, we found a broom closet, 9 x 4 feet, with a sterilizer at one end.

There was a lot of work to be done to make that space workable, but the engineer said that it was feasible. However, the matter of cost did not come into discussion.

Before I left, Dr. Wilkins asked me to hold up my decision for Boston until he could get some information he needed. Back for two-weeks in Salt Lake City, I got a phone call from Lawson; the work could be done, it would take three months. Then he asked if I would come back to Hopkins. After one more week, I called Lawson to tell him I accepted his offer and would come back in October 1955.

## Return to Baltimore (October 1955)

The friendships I made with my colleagues at the University of Utah were memorable. The social life was wonderful. There were group trips to Sun Valley and the Grand Teton Mountains, to Yellow State Park with a stay at the Inn. The expeditions to the southern parks including Mesa Verde were a lot of fun, as were trips to Reno and San Francisco for meetings. Despite all this, I had a feeling of being isolated. San Francisco is 750 miles from Salt Lake City and the East Coast is 1700 miles away. So after three wonderful years in Utah, I was ready to move back to the East Coast. I thought I was going to Harvard, but I ended up at Hopkins again.

Having acquired lots of things to carry with me, I decided to buy a second hand Chevrolet. It was not a beautiful car, but I believed that it would carry all my junk and books. I also filled up a large trunk with my belongings and shipped it by train to Baltimore. It was so heavy that I managed to rupture a dorsal spinal disk. Bob Martin helped me to move it to the station.

There were several suppers and parties to say goodbye to the many colleagues, coworkers in the lab, the Samuels, and the lovely Wasatch Mountains.

But eventually, I took to the road. Lytt Gardner had organized a meeting in Syracuse, New York by. So I planned to drive to Lake Ontario before heading south to Baltimore.

I was quite naïve about the distances in the USA. The way to go was simple: route 80 to south Chicago and then route 90. It took me several days with a stop at Cheyenne. I did not realize that the state of Nebraska was so long: a very monotonous trek with hills after hills. I used my radio for company but on Saturday, there was only college football. I had to listen to one game twice, as it was repeated later in the day. But finally, I made it to Syracuse.

There, I found all my old friends from the east coast: Dr. Wilkins, Bongiovanni, Crigler, Eberlein, and the whole psychohormonal group: John Money, the Hampsons, and fellows. Lytt Gardner was now director of Pediatric Endocrinology at Syracuse University. I was glad to see all of them.

The meeting was very exciting. John Money was very prominent in all the discussions relating to sexual abnormalities.

After the meeting, Dr. Wilkins told me to take route 81 to Harrisburg and then 83 to Hopkins, and invited me to stay at his home, until I could get resettled in Baltimore.

Searching for an apartment of my own, I quickly found one on the 2^nd^ floor of a private home on Schenley Road. One entered the apartment through the living room. There was a large bedroom, with a small balcony, a very small kitchen, and a tiny bathroom. It was furnished but minimally. There were some red plastic curtains at the windows, which gave a bad impression at night when my lights were on. I bought a second hand radio and this is where I lived for the next five years.

## Back to Hopkins

I enjoyed finding colleagues who I already knew. The fellows were Jud Van Wyk and Melvin Grumbach. John Money was an old friend. The technicians were still Mary-Ellen Crafton and Betty Youngblood. I was then an Assistant Professor.

At the north end of the 5^th^ floor, I found Dr. Victor Najjar, who worked in the same space as Dr. James Gamble and Dr. Jim Sidbury. In Boston, I had met Gamble’s father, an expert in electrolyte equilibrium in newborns and infants. Both Jims offered to help get my trunk at the station. Jim Sidbury was a real Southerner from North Carolina. A real friend, he called me “Cousin Claude” and I called him “Cousin Jim.”

Dr. Wilkins told me that I had to pay a visit to the head of the Department of Pediatrics, Dr. Schwentker. So I made an appointment with the secretary. When I entered the office, I noticed immediately a vertical wood stick on the side of the desk of Dr. Schwentker with a magnificent, huge bird of many colors. I shook hands with the pleasant looking man across his desk and he asked me to sit down in front of him. At that point, there was a big noise of feathers flying up in the air. And as Dr. Schwenker sat down, the parrot flew to rest on his right shoulder. I was very perplexed when the Chief of Pediatrics welcomed me into the department. I was not quite sure if I had this conversation with the man or the bird. I said thank you quickly and left his office, flabbergasted. Of course, I reported my adventure to Lawson, who smiled a little and told me about Dr. Schwentker’s struggles with alcohol abuse.

I also found several French-speaking doctors at the Baltimore City Hospital (now called Bayview). In Medicine, there was Ives Boennec and Francis Bazin with his wife Jeanine, a nurse. In surgery, there was Felicien Steichen from Luxembourg, the nephew of the very popular photographer Edward Steichen, the author of the famous exhibit “The Family of Man.” Their group decided that as I was a little older, I was to be their “uncle” and they called me “Tonton Claude.” At that point, I had obtained the family designations of both “cousin” and “tonton.”

Not everything can go as anticipated. My poor old Chevy had crossed the USA brilliantly but it expired shortly after arriving in Baltimore. I asked a mechanic to look at it but his diagnosis was terrible. I would have to change the motor completely. Basically, the car stayed in front of my apartment, incapable of moving. At that stage, I did not have the cash to buy another car. Lawson decided that he could make a little detour and drive me to and from my apartment on Schenley Road every day. This started a series of twice daily twenty-five minute discussions with Dr. Wilkins.

## Daily commutes with Dr. Wilkins

It turned out that the daily trip from my apartment to Hopkins Hospital in Dr. Wilkins car was a great way to have very personal conversations.

### Conversation 1: Dr. Wilkins and his family

One day, Lawson would start talking about his family life, how Lucile was good to him and tolerant of his idiosyncrasies. She had been very supportive at the time of the accident and death of Skippy. She had encouraged him to go back to carpentry. She helped him to go on his gardening, which was very comforting to him.

I learned that Lawson was quite fond of his mother-in-law, Mrs. Mahool. However, he did not understand how a conservative person like her could vote Democratic instead of Republican like him, just because she was a southerner. He was not as fond of his brother-in-law, Tom Mahoul, who having never married, still lived with his mother.

Dr. Wilkins would tell me that Skippy went to Gilman School but had some problems with schoolwork. Yet, he was a very attractive young man, very good in sports, and had a great deal of social grace. He probably had a mild learning disability. Although Lawson thought that Skippy might not be able to make it in medical school, he thought that his son would have been great in business or law. His daughter, Betsy, was an excellent student but she was not interested in medicine as a career.

### Conversation 2: my education in France (1936-1950)

On one occasion, Lawson wanted to know about my schooling in France. I told him that it had been quite complex. First, I went to Ecole Carnot in Lens (Pas de Calais) where I was a good enough student but did not work very hard. After my mother died, I went to live with my mother’s sister who had been married ten years with no children. So she and my uncle who was also my godfather were pleased to take my brother Michel and me. Her husband was my godfather, but he was also director of the small college of Rethel (Ardennes). My aunt played piano and taught music at the school. The move changed my attitude toward schoolwork. I continually competed for first of the class with a child named Guy Voillemin. Then in 1939, the Second World War started. We were obliged to move from Rethel to our evacuation site, close to La Rochelle at Fontenay-le-Comte (Vendée) where I went to the College François Viete.

In the fall of 1940, the Germans had invaded France and Rethel (Ardennes) was in a “zone interdite,” but because we lived there, we were permitted to return. When we arrived, we found that many houses of the city had been destroyed. Fortunately, our house survived; there was only a hole on one side above the garage that was later repaired. However, a German officer was living in our house. When we rang the bell, a soldier opened the door, and called his officer. We discussed our problem with the officer who agreed to let us in. But he would take the salon (our living room) as his office, the dining room for his bedroom, and he demanded access to our kitchen. This was an awkward situation for six months until the officer was called to the Russian front. Lawson wanted to know how we could live with the German solider. I explained that compromises were necessary, if we wanted a roof over our heads.

For school, I had to go to the Lycée de Reims and I lived the first year with a large family in their big house. The winter of 1939-1940 was absolutely miserable and I can say that I know what it means to be hungry. The harvests had been lost, the cattle destroyed. We had tickets, 25 grams of meat per week, no milk, and no bread. By the spring of 1940, people began to use their gardens to cultivate potatoes. We had a hen in our garden, which gave us an egg from time to time.

In 1941, I passed the first part of the baccalaureate and the second part in 1942. I rented a room from a very nice lady, Madame Moraux, who lived with her 12-year-old grandson Christian. From our 3^rd^ floor apartment on 44, Rue Jean Jacques Rousseau, I first saw bombs falling from allied planes on the airport of Reims! It was strange.

In the summer of 1942, I had to decide what my career would be. I was very good in mathematics and considered competing for what the French call “les grande écoles” like Polytechnique. But I thought that maybe I was not good enough. My best friend Huber Pautet felt strongly about medicine, so I decided to do it too. Reims had a school for the preparatory year, “physique, chemistry, biology” or PCB. At the end of that year, there was an exam and only half the students could start the first year of medicine. At that time, Reims provided only the first two years of medical studies.

On January 1, 1945, after the “liberation,” I was called into the army, where I ran an infirmary on the front in Alsace.

As the armistice was signed on September 1, 1945, I was permitted to go back to school for my third year at the Medical School of Paris. We rotated through various hospitals in the city. I took my Pediatrics rotations at the Hopital Necker-Enfants Malades, Rue de Sévres. My mentors were Dr. Julien Huber, Soterios Briskas, and Robert Debré. This rotation convinced me to specialize in Pediatrics. In 1949, however, I spent a short rotation on a ward (about 20 beds) full of children with tuberculous meningitis for whom no specific treatment was available. This shocked me. At that time, I began to realize that doctors had to do research to solve such medical problems.

Of interest, at that time, I attended girls with Turner Syndrome. I obtained some of my information from your papers, Dr. Wilkins. I have to admit that I had no idea that I would work someday with the smart physician who had published these papers.

Lawson Wilkins explained how he was able to better understand the pathophysiology of the Turner Syndrome: with the help of Dr. Howard Jones, the gynecologist, he found that these girls had no ovaries. He also studied their multiple congenital malformations including heart, kidneys, webbed neck, and poor stature and growth. It was strange for me to remember the time when I was reading the paper of Wilkins L and Fleischmann W of 1944 on “Ovarian Agenesis” while seeing such patients in Paris. This great physician was now sitting next to me and was my mentor driving me home.

### Conversation 3: Dr. Ernest Schaffner and the French Resistance (1939-1945)

On one of our trips, Lawson wanted to know about the French Resistance in the Second World War. I told him that I was not officially a member but I worked with Dr. Ernest Schaffner, a 40-year-old friend of my family who I found out later was in charge of a resistance medical network in the region.

Dr. Schaffner was born in 1901, in Strasbourg, as a German citizen since Alsace had been annexed by Germany after the war of 1870. His father was the conservateur of the beautiful cathedral of Strasbourg. He spoke an Alsatian dialect, but also knew French and German. Since 1918, Alsace/Lorraine again belonged to France. During his years of medical school, he contracted tuberculosis but recovered after several stays in a sanatorium. In the late ‘20s, he was named Chief Physician of the “Dispensaire antituberculeux d’hygiene sociale” of the coal mines of Lens/Liévin. He organized a hospital and several infirmaries in the region. He was a physiologist who studied silicosis of the coal miners. Many had tuberculosis as well.

In the summer of 1943 and 1944, Dr. Schaffner accepted me to work with him. He was willing to state that he needed me, and that avoided my being sent to work in Germany. He had known my mother and was a friend of my father. I followed him everywhere as he worked at the hospital, the dispensary, and making home visits. There was not any real treatment or antibiotics at the time. Dr. Schaffner used pneumothorax and followed the effects on the lungs by X-ray. This was successful in a few cases of tuberculosis but not for silicosis.

When I went to work with him, he had been in practice for many years. By that time, he had lost several fingers of his left hand and one finger on the right. He was a victim of the X-ray, like its inventor Dr. Marie Curie.

Now, he was using gloves for protection and made me do the same. We visited the dispensary in the morning, and after that the hospital where patients were cared for by nuns. Sister Martine would have a sandwich and cup of coffee for us.

Dr. Schaffner was adored by the patients, their families, and the nurses. They called him the “Apostle of Lens.” When we went to a café for a drink, he would never be charged for it. At the patients’ homes, there was always something for us to eat or drink. The devotion of Dr. Schaffner to his patients was equaled by the devotion of the patients to their doctor, whom they saw as a father.

Because he spoke German, he could communicate easily with the German authorities. He was respected by the Kommendantur, as he was very important to the health care of the coal miners, as the Germans used the coal for their industries.

I also admired Dr. Schaffner and asked if I could help him in any way. One day he asked me if I could deliver some letters in Paris because he did not trust the post office. I accepted rather innocently, not realizing that I had become a “carrier.” I would be given a ticket for the train to Paris, and I would deliver two or three letters each time. When I was in Paris, after the delivery was made, I would visit my cousins Jeanine, Fabienne, and Edmée ,who all worked at the Service des Eaux de la Ville de Paris. I thought this was fun, not realizing that if I were caught, I could be sent to a concentration camp. Dr. Wilkins wanted to know if I was scared. I guess I looked fairly young and innocent when I was 20 years old and in fact, did not know what I was doing.

### Conversation 4: the English pilot at the French brothel among German soldiers (August 1943)

Another story I had to tell Dr. Wilkins about was my other experience in the French Underground. At the end of one day, Dr. Schaffner told the nurse and me that we had to go immediately to the brothel.

I should give some explanation: I knew that a certain café was a house of prostitution. Like everyone else in town, the madame in charge knew Dr. Schaffner. The prostitutes were followed at the Dispensaire d’ Hygiene Sociale. This is where I became an expert in taking blood samples and infusing IV therapy for syphilis.

When we entered, we were in a large room, full of smoke with tables and German soldiers and French girls drinking at the tables. The madame took us into a little room, closed the door and asked us what she was to do with the English pilot that had just arrived.

As it turned out, the brothel was the place to which the underground would send allied airmen who had been on their way to bomb Germany when they were forced to parachute into Holland and Belgium.

Dr. Wilkins wanted to know what we did with the English pilot. I was the only one in our group who had had four years of English classes, so I was the one to check on the man. I was also asked to check his underwear to make sure that they did not have German labels. I could verify that the labels were from Utrecht, Holland.

I was told that the man would be sent to the next relay, on his way to Portugal and then on to England. I have no idea if he made it.

Dr. Wilkins and I further discussed the occupation of France by Germany. The French tried to survive the best they could. The madame and the prostitutes would entertain German soldiers. Yet, at the same time, they were involved in the Resistance. This was hard to explain but it was a fact of life.

Dr. Schaffner did not run into trouble with the German authorities, which was something of a miracle. After the war, he went to visit his friend Dr. Albert Schweizer, the Nobel Peace Prize winner, in his renowned Hospital in Lambaréné (Gabon). He died at 65 years of age, the result of radiation exposure. Lawson agreed with me that Dr. Schaffner lived an unusual life.

### Conversation 5: Dr. Wilkins and World War I: (Figure 4)

**Figure 4:**
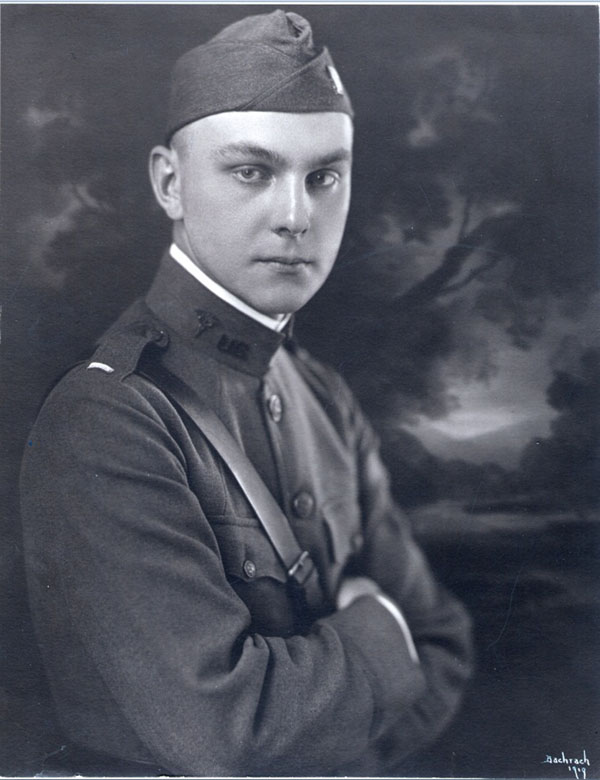
Lawson Wilkins in Johns Hopkins Medical Unit—1917

On one of our trips, Lawson told me how he had been involved in World War I. When the USA entered the war, the Hopkins Medical School decided to send to Europe a base hospital consisting of staff members of the school along with medical student volunteers.

In May 1917, a large group of medical personnel was formed under the direction of Dr. JMT Finney as Base Hospital #18. The group was made up of physicians with all specialties represented. There were thirty-two 3^rd^ year medical students including Lawson, pharmacists, nurses, physical therapists, dentists, administrators, laboratory technicians, secretaries, carpenters, and cooks. In addition to all these people they sent along a large amount of supplies including beds, surgical equipment, and drugs. The Base Hospital #18 boarded a ship in New Jersey and sailed to France, on June 14, 1917.

The conditions on the ship were not the best, and Lawson reported that much of the personnel became seasick. There were so many people on the boat that they could not come on the deck together. They had to rotate in order to get fresh air. Because of the presence of German submarines, the boat took a torturous route which took 14 days before arriving at St. Nazaire, the French harbor at the estuary of the Loire River.

They were rapidly moved to Savenay about 10 kilometers north of St. Nazaire; there was a large hospital base there. Savenay was a lovely little town and the native French people were very friendly. Lawson had been able to travel a little north of Savenay, visiting Brittany, which he loved.

After a month at Savenay, they took an eleven hour train ride to reach the Vosges in Lorraine. They debarked at Bazailles sur Meuse, a village of farms with about 500 inhabitants. The Base Hospital #18 settled near the Chateau de Bazailles: this was a 1000 bed unit which opened on July 26, 1917. Six other Bases, each of about 1000 beds, were also established on both sides of the Meuse River.

The base, according to Lawson, was almost four miles south of Neufchâteau, a small town of 7000 inhabitants. The country was very lovely with the Vosges hills and beautiful woods.

Lawson and his schoolmates played the roles of interns, anesthetists, and lab technicians. However, they also had to do all sorts of work like cleaning the latrines and helping in the kitchens.

They cared for a lot of sick soldiers as well as many wounded men. They received casualties from various fronts: Verdun was 70 miles north, Nancy 40 miles east. The “Second Battle of the Marne” and the Bois Belleayre near Fère-en-Tardenois and Reims was not far. At the medical center, they had 50,000 patients and 800 deaths.

The American army sustained very heavy losses, a total of 50,000 men – most of them killed at the front – and 200,000 wounded. Of course, the total allied losses were about 2,500,000 men –the same number as the dead Germans/Austrians.

Lawson wanted me to know that from Savenay, he had gone 15 miles north to Vannes, a lovely old city on the coast. There he was invited by French people to come and participate in their meal. Lawson was willing to say that there were also three lovely young ladies. He did not go into further detail, nor did I request more details. But it is a fact that Dr. Wilkins had become a lover of French culture.

### Conversation 6: the “Houses of Tolerance” at Savenay during World War I

On another trip, Lawson told me about one unusual aspect of the Franco-American alliance. The city of Savenay was close to the harbor of St. Nazaire and it had many “houses of prostitution” open to sailors and visitors. The hygiene of these places was very poor and there were numerous cases of venereal disease reported.

After the debarkment of the first groups of American troops, the military authority became concerned and General Pershing made the houses of tolerance off-limits for the American soldiers. It would appear that the mayor of the city complained. It is said that it went to the President of France, Georges Clemenceau. After long and arduous discussions, the control of designated houses would be given to American medical authorities.

I asked how this sexual conflict was resolved at the levels of the high authorities. Lawson was not sure. When Base Hospital #18 was closed on January 20, 1919, Dr. Wilkins returned to Savenay. He thinks that there had been a solution to the problem.

On February 20, 1919, the personnel of Base Hospital #18, including Wilkins, was back in New Jersey. Lawson was trying to find a position of intern in the US. Despite that late date for finding a position, he managed to find a place at Yale!

### Conversation 7: how Lawson Wilkins came to develop the field of pediatric endocrinology

On September 1, 1939, World War II started in Europe. The USA joined the conflict after the attack on Pearl Harbor on December 11, 1941. Wilkins said he was too old to join the army. He became very busy, covering the practice of other pediatricians. His routine was early home visits to patients. Then it was the hospitals, both Hopkins and Union Memorial Hospitals. This was followed by his practice at his office, 1014 St. Paul Street. He finished his days very late; Lucile would prepare his supper, and he claimed that he needed a stiff nightcap, usually rye alcohol, to carry him to sleep until early the next day.

Dr. Edwards Park, chief of Pediatrics at Hopkins, had offered him a full time position that Lawson refused via letter on December 17, 1943, saying, “For years, I have found myself torn between the things I would like to do and those I have to do.” Lawson felt a duty to his practice and often at night he would consider the problems of the research clinician.

On January 10, 1944, Dr. Wilkins was seeing patients at Union Memorial when a nurse came to tell him that his son was in the hospital, victim of a car accident. Lawson rushed to Skippy’s side, but found him dead. This was a terrible shock!

I think that horrible accident helped him to accept the position of Associate Professor in 1946, offered by Dr. Park’s successor, Dr. Francis Schwentker. At this time, he started seriously collecting data on pediatric endocrine patients. During the following four years, Lawson built the foundation of his famous book “The Diagnosis and Treatment of Endocrine Disorders in Childhood and Adolescence.” It was published by Charles C. Thomas in 1950, thus beginning the new field of pediatric clinical research.

### Conversation 8: the end of daily rides with Lawson

Eventually, I figured out the means to pay for a new car. Dr. Wilkins advised me not to buy a second-hand car. He thought that a Chevrolet would be a good deal, and I took his advice.

Dr. Wilkins knew the manager of the local Chevrolet dealership. He managed to get me a low price on my new car and even some money for my broken car. Of course, my new transportation made life much easier for both Lawson and me.

This was marvelous. Unfortunately, we lost the opportunity to have personal conversations twice daily. However, I still went with Dr. Wilkins to the many lectures that he gave at the Naval Hospital and National Institutes of Health in Bethesda, the Baltimore City Hospital, and Union Memorial Hospital. Lawson was in great demand and very generous with his time, but he liked to have company and it was me!

## Social life in Baltimore

Dr. Wilkins loved parties and Lucile was very accommodating. While he was temporary Director of the Department of Pediatrics after Dr. Schwentker's death, he had parties for the house-staff at his home, which took place at least twice a year, at Christmas and in the summer. These were occasions for lots of food and even more drinking. I remember an occasion when Lawson had to take a nap in the grass of the backyard. But before that point, there was a lot of singing; one member of the house-staff played his guitar and a good time was had by all.

During the winter, there was opera at Lyric Theater. I was invited on several occasions. For the opera, Lawson would smoke as it was a social occasion.

As much as Lawson liked Rigoletto and Carmen, he loved the Gilbert and Sullivan operettas. I also was a member of the party and after the show, we had to review the major songs before separating for sleep. Often, he managed to bring up the fact that Skippy loved that specific song and that he had a better voice than his father.

On Sundays during the summer there were trips to the Chesapeake Bay. Dr. Wilkins had a small boat, about eleven feet long, that he manipulated like a real captain. I think that Lawson had cruising vacations with some friends using a large Chesapeake oyster workboat. These two week adventures, I had been told were a lot of fun.

**Figure 5:**
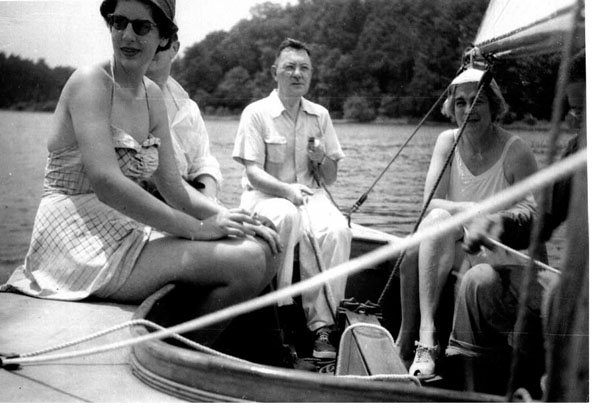
The Wilkins family with the Captain of the sailboat. (1955)

**Figure 6:**
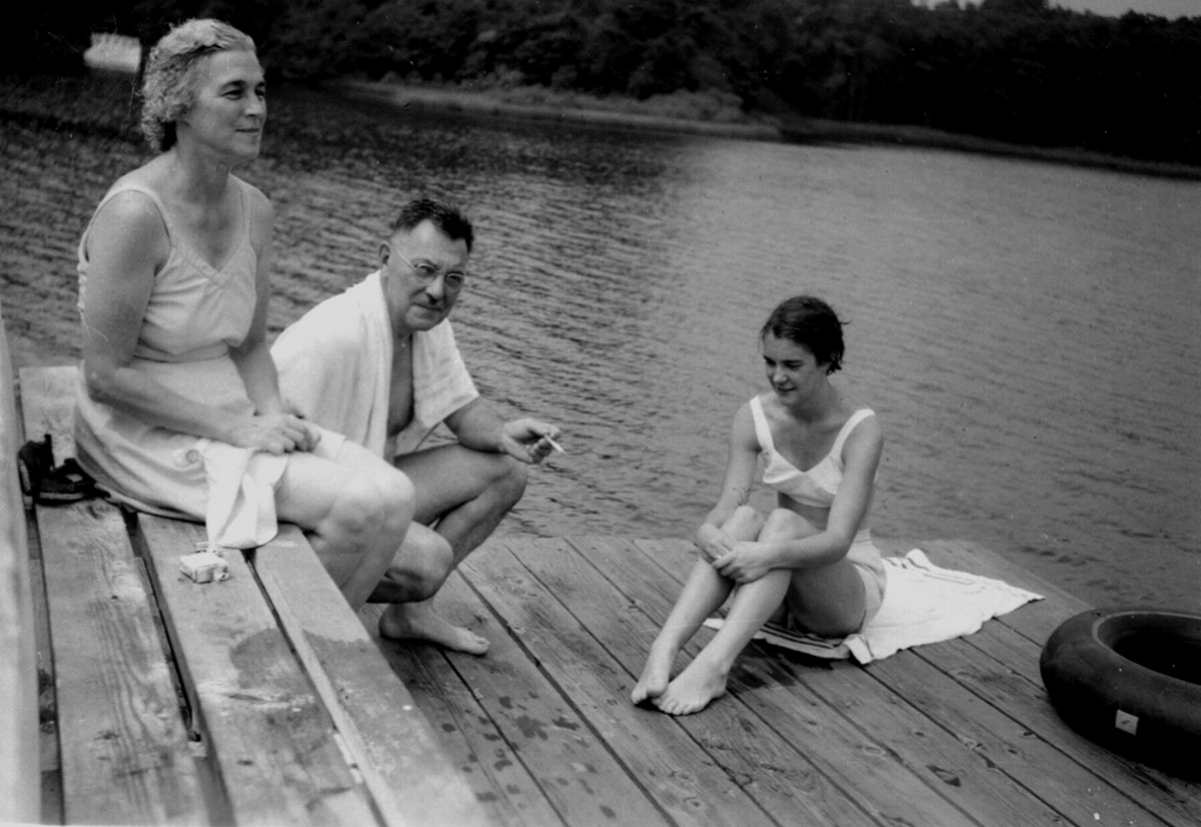
Lucile, Lawson, and Betsy Wilkins after a swim. (1955)

The little sailboat that Lawson shared with me was a miniature dinghy, but Lucile, Betsy, and I would receive orders from our "Captain." After a while, Lucile would get the bag with food: fried chicken, potato salad, cucumbers, and cold tea. After returning to the dock, there would be a swim, songs, and a more vigorous drink prior to the return home. (Figure 5, 6)

In July 1958, the Wilkins family took a three-week vacation on Cape Cod. I went to join them for a few days. The chalet was right on the beach. We swam, went fishing, and at night, we played very serious games of bridge. I was the partner of Mrs. Mahool, who was a very smart player. The team of Lawson-Lucile never won, and Lawson was most unhappy. An extra glass of rye alcohol was necessary to console him. A compensation for Lawson was I could give him news of the patients he had left in Baltimore ten days before who I had seen the day before I arrived.

The winter of 1958, Lawson told me he was going with Lucile to Bethlehem, Pennsylvania to see Emilie, his sister. I was invited to join them. The main attraction was the yearly concert by a fantastic choral and orchestral group at the Moravian church. The program was all Bach, an exceptional occasion. As the local inhabitants were mainly German immigrants, the songs were in German. The concert lived up to the high expectations that Lawson had set for it. It was absolutely magnificent. On the whole, it was very impressive!

I must not forget gardening. This was an important recreation for Lawson. He thought that I did not take enough exercise and he would invite me to come to help him. There was grass to cut, bushes to trim, flowers to plant. It was also an occasion to have a talk with the neighbor, Mr. Allnutt. And of course, as it was hot, we had to have a drink. Lucile would always find some food to go with drinks and we had a lot of fun.

## Reunion of members of the class of 1918 of Johns Hopkins Medical School

On the occasion of their class reunion dinner, Dr. Hugh Morgan from White Bridge, Tennessee gave the following address:

“In recognition of the essential roles Wilkins played in the United States Army in World War I, certain facts should be recorded at this Hopkins dinner honoring Lawson.

Wilkins was the youngest in years and in heart of the thirty two intrepid soldiers recruited by the Hopkins Base Hospital #18 from the class of 1918. This unique recruitment benefitted enormously the Baltimore residue of the class since it removed a large number of highly talented but odd and difficult characters. It also benefited Base Hospital #18 by providing it with, at one and the same time, military and academic personnel capable of becoming bathers, delousers, bedpan hustlers, duck shooters, grave diggers, and honey dippers. By on-the-job training, Wilkins eventually qualified as “expert” in each of these assignments. By his own admission he surpassed the other students in his performance and devotion to duty. Because of this, he achieved almost immediately the military grade of private 1^st^ class, and furthermore, he was successful in retaining this title, without demotion, throughout his entire career as a student-soldier. All of his classmates agree that wherever he was and in whatever assignment he functioned, Wilkins worked incessantly and successfully.

Our remaining observations have to do with Dr. Lawson Wilkins, Professor of Pediatrics, the Johns Hopkins University.

The class of 1918 recognizes and proudly acclaims Lawson Wilkins as, far and away, its most distinguished member. Clinician, clinical investigator, teacher and author, a professor in his own superb and inimitable manner and, therefore, in the true Hopkins tradition.

On this memorable occasion Lawson Wilkins’s classmates acclaim him and wish for him many more productive happy years.”

Hugh Morgan

## Dr. John Eager Howard: a colleague, friend, and physician of Lawson Wilkins

**Figure 7:**
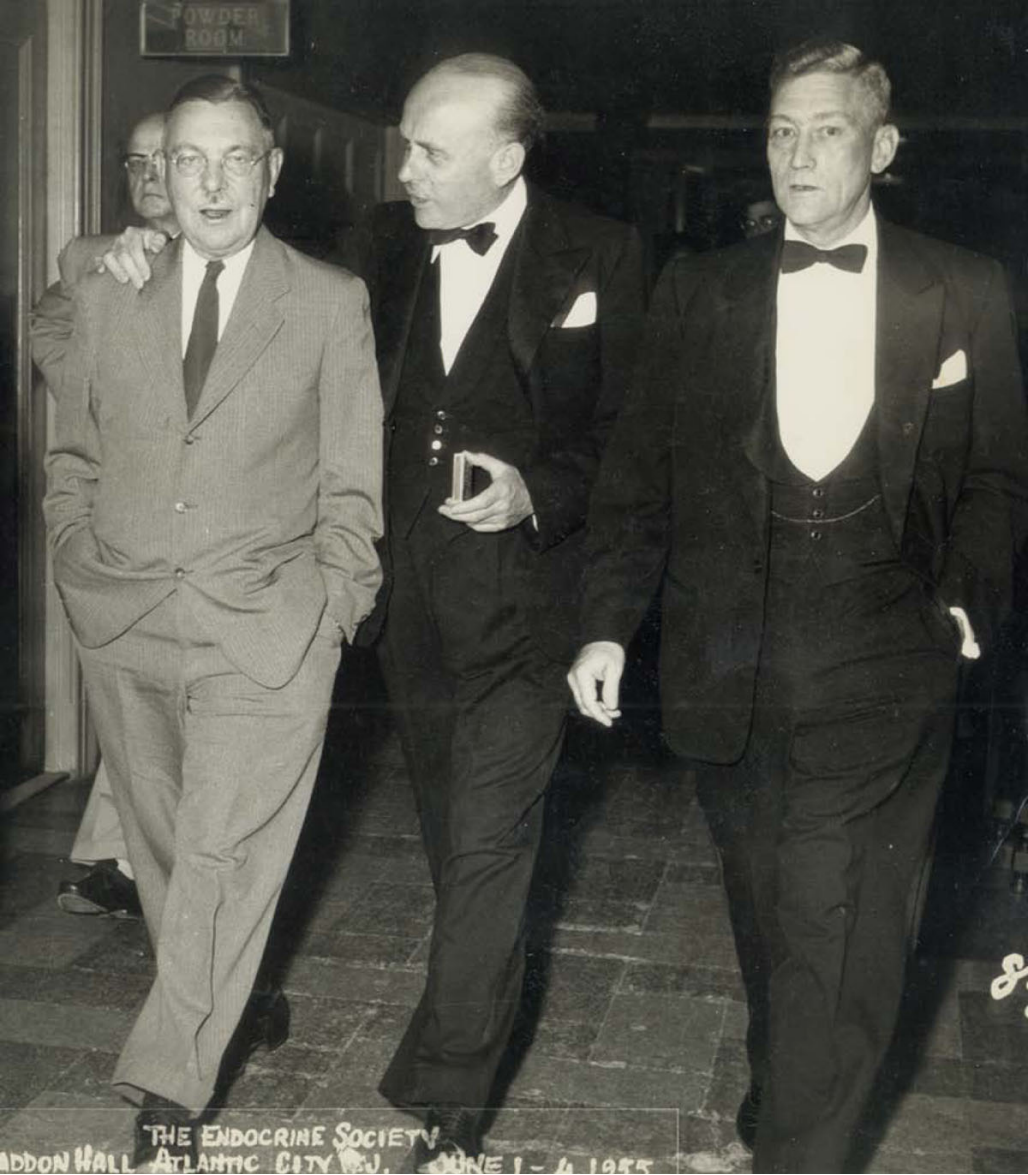
The Endocrine Society Meeting, Atlantic City, NJ. from left: Lawson Wilkins, Douglas Hubble, and John Eager Howard. (June 1-4, 1955)

It is important to introduce Dr. Howard when writing about Dr. Wilkins. Dr. Howard represents the aristocracy of Maryland. His ancestor was a general in the Revolutionary War; the statue of General John Eager Howard on horseback is placed right in the middle of Mount Vernon Square in Baltimore City. Dr. Howard reports that on one occasion, his friends put him onto the statue, and he had to explain to the intervening policeman that the statue was of his ancestor. (Figure 7)

Howard lived in a lovely house in Hunt Valley. He was a professor in the Department of Medicine, and was chief of the adult Endocrine Clinic. He held a weekly clinical conference in the Hurd Hall Auditorium, where adult and pediatric patients with difficult or rare diagnoses were presented with Dr. Howard directing the show and tell.

The audience consisted of approximately 100 physicians. The residents who presented the patients were intimidated by Dr Howard, and tried hard to be complete and clear. If not, Dr. Howard would be very critical and ridicule the presenters who made mistakes.

One day, I was on the elevator with Dr. Howard. I thought it would be polite to ask, “How are you Dr. Howard?” He did not respond immediately and then looked straight at me and said, “Why do you ask?” Fortunately, by this time I had arrived at my floor, and did not have to answer this question.

After Dr. Wilkins’s death in 1963, Bob Blizzard and I organized a meeting of the past fellows and their colleagues (a group of over one hundred people). The reunions took place every two years, 1965, 1967, 1969, and 1971. At the last meeting, the Lawson Wilkins Pediatric Endocrine Society was created.

At each of the meetings, a big scientific program was organized. It included a supper with drinks to honor Lawson.

On April 29, 1969, we had our dinner together on the top floor of the School of Hygiene. The food was quite good and we had a great deal of wine that I had bought for the occasion. I had asked Dr. Howard if he would say a few words after dinner. I believe that he was quite touched by my request and he gave the following speech:

“The scientific sessions of the day have passed; we have regaled ourselves with delicious viands and the fruit of the vine. The time has come for more mellow thoughts in our recollection of him in whose memory we are foregathered.

To have been selected to make some remarks on Lawson Wilkins under these circumstances has induced in me rather vigorous emotions, and a consequent fear of inability to do justice to the occasion. For we were close friends as well as enjoying the relationship of co-workers in a medical vineyard from its earliest days of respectability and even before it was respectable. I refer, of course, to the field of clinical endocrinology. Furthermore, there was the doctor-patient relationship; and, in all of these contexts, a love and admiration for the man continued to grow and flower.

I never knew much about Lawson’s youth, medical school years or his time spent in the Army during World War I, except that he distinguished himself by devotion to duty and was highly thought of by his seniors in the Hopkins Medical Unit – for example, by Dr. J.M.T. Finney and Dr. Walter Baetjer, who always spoke of him to me retrospectively in the most glowing terms.

You did not come tonight to hear rhetoric of what I have heard of Lawson from others; I have rather tried to record my memories of him as I saw and recall our times together, and there were few dull moments. My first non-casual meeting with Lawson took place, as I recall it, under the aegis of Dr. Park, who had kept telling me that I must know and work with this extraordinary man. The youthful doctor’s talents had become evident to Dr. Park early, and he still believes one of his very greatest achievements was in this recognition and in the part he was able to play in the fulfillment of Lawson’s great career in academic medicine.

On the day of our meeting arranged by Dr. Park to discuss some of the beautiful observations which had just been made on skeletal effects of thyroid deficiency in very early life, made with meticulous care and documentation while still actively engaged in practice, I recall distinctly being much less impressed by the beauties of the studies than by the vitality of the man. In response to Dr. Park’s gentle knock, the rafters fairly reverberated to the booming voice that urged us to come in; and the sound must have been audible in Washington. This characteristic of his was never out of evidence during our long association and made it impossible for me to exchange confidences with him except in a car or a sound-proof room. Lawson’s whispered asides, when, for example, he disliked what a speaker might be saying or the manner of its presentation, could at times cause consternation, for his “that fellow is putting out pure hogwash” might be heard all over the room. But I should hasten to add that his comments were rarely uncomplimentary, for an immense generosity toward the failings of others was one of his most endearing qualities.

The very nature of the man exuded friendliness. Dr. Park has remarked about the late Dr. J.M.T. Finney that a stranger shaking his hand could feel at once the warmth that radiated from him. Lawson, too, had this radiating warmth; and one felt instinctively that his desire was to be kind and on your side if it was in any way in the cards for him to do so.

Another outstanding characteristic of Lawson Wilkins was his patent genuineness. You could literally see right through the man; “he wore his heart on his sleeve.” This, together with expertise, caused almost adoration in the parents of his patients, and no doubt among the patients too. Prior to giving up his practice, it was his habit to start work at eight o’clock in the morning, not coming home until eight or nine o’clock in the evening, when Mrs. Wilkins invariably had dinner waiting for him. Between the hours of 12 and 2 A.M. he worked on the charts of his studies, meticulously designed and beautifully executed, which became the hallmark of his published investigations. The physical endurance of the man was greater than in anyone I have ever known. Dr. Park has told me that he had offered Lawson charge of the Epileptic Clinic at Harriet Lane, which was declined for lack of interest, and the lack of scope of the problem. But when it was proposed that he head the Pediatric Endocrine unit, this post was accepted. Here he was so successful and his work so original that, with Dr. Park’s continued prodding, Lawson finally was persuaded to take a full-time appointment provided by Dr. Schwentker; and his practice was sorrowfully abandoned.

Early in our days of collaboration and friendship, it was, I believe, his suggestion that we meet together once a week and see and discuss some sick persons. If anyone wanted to join, so much the better, for – from their questions or remarks – we might pick up some ideas to pursue which might enlighten us. Hopefully also there might accrue therefrom something which might assist the very young and groping efforts then extant to study the field of glandular abnormalities. The operators in this field were at that time demeaned by the rest of the profession to the category of outright quacks or mountebanks. And this categorization was indeed not too far from the truth. The Society for the Study of Internal Secretions used to meet at Atlantic City just before the boardwalk might be filled with the distinguished members of the Association of American Physicians, in whose eyes we hopefully wished to appear in the best possible light. We used to take off our badges on leaving the endocrine meetings before taking our boardwalk stroll. There are probably few or none of you in this room who remember those days, as participants at least. Some of the really corny articles of the then Society for the Study of Internal Secretions are gems of science which might curdle your blood. Small wonder that Fuller Albright, Allan Kenyon, John Browne, Lawson Wilkins and their like removed their badges before being seen by the Thayers, Longcopes, and the Parks.

As a patient himself, Dr. Wilkins left much to be desired. On the first occasion when this relationship began between us, Lawson, then in his early forties, was lying on a couch in great agony from an immense flaming red toe. “Do you think this damned thing is the gout? My father had the gout, you know; but don’t touch it if you value your life.” His annoyance with the effects of colchicine was nearly as great, or greater than that from the pain in the toe, though it cleared his podagrous symptoms magically. This was before the days of uricosuric agents or enzymatic blockers of uric acid formation, and the advice was required to be much more careful of certain liquid and solid materials of which he was dearly fond. It was characteristic of the flamboyance with which he went through life that he religiously ignored my advice in this regard. It also may indicate something of the inner and hidden strength of the man, for he had only two or three minor recurrences during the subsequent 30 years. On many occasions he seemed to gain surreptitious pleasure by looking directly at me across a table and waving his glass or his fork when proceeding directly contrary to my medical orders.

In Lawson’s later illnesses, the pattern of resistant behavior did not change. After his accident out near Frederick in his tiny car, he steadfastly refused to accept what appeared to me an obviously broken neck (fracture of a cervical vertebra) and constantly fretted with the relatively minor interferences with his activities which were imposed by the traction and, later, Thomas collar. He drove a car again far earlier than deemed wise by his orthopedist; but despite my pleas that another even minor accident at this time might incapacitate him for all time, he did it and, characteristically, got away with it.

The same fierce hatred of disability characterized his reaction to a very serious coronary occlusion. Added to the burden of immobilization which alone would have sorely tried him, was the fact that at the time he was ardently courting his second wife, without whose powerful aid I suspect we could never have kept him quiet until such time as his infarction had healed. But the robust joie de vivre returned, and Lawson traveled widely abroad and even climbed mountains for several years before his second coronary finally carried him off. This area of necrosis caused by this first occlusion was so massive that the pathologists could not believe he had been able to do all the things he did right up to his final illness.

But by far the greatest of Lawson’s monuments (and no tribute of mine can approach its magnificence) is the presence of you men and women at this dinner. Betsy is a monument in herself, and no mean one. But I refer also to you, his students, who, without undue flattery, make up the core of most distinguished pediatric endocrinologists in the world. And you wouldn’t be here if one and all of you were not proud to be called “Lawson’s boys.” Few men have left behind them so distinguished a group of pupils and colleagues, all crucially influenced in your careers and greatly indebted to that extraordinary man.

It is happy for me that you permitted the Howards to join you and present some of my reflections on a long period as friend and colleague of your guide and mentor. And I am also deeply conscious that some of the encomiums that have come my way have been directly due to my privilege of being co-worker with Lawson Wilkins and, for that matter, with most of you also.

## Work schedule and financing of the fellows at the pediatric endocrine clinic

Wilkins’ fellows had very defined duties. As time went, it was organized that they were spending their first year of fellowship in the clinic. This was a clinic every morning on weekdays, and there were mainly outpatients and a few inpatients. The charts were dictated by the fellows and then checked by the “chief.” In that first year, they were encouraged to consider a clinical study, which could bring them to a laboratory study on their second and third year. At all times, everybody attended the “Saturday Clinic,” previously described.

There were also weekly reviews of literature, every Wednesday evening. Usually, we would go out for supper at a Chinese restaurant “Me-Jon-Lo,” or the “Steak House.” Then we would come back for a couple of hours in the library of the Harriet Lane Home.

At the same time, I had been asked to join NIH meetings to organize the postdoctoral grants. The other colleagues and I agreed that the three year Hopkins plan could be adopted.

In 1959, Lawson took charge of our first training grant (2G-335) followed by a long range grant starting in 1960 (2A-5219). These covered the salaries of five postdocs.

In 1951, Dr. Wilkins had applied for an NIH research grant. When the NIH had started after WWII, there were three branches: cancer, cardiovascular, and arthritis and metabolic diseases. At that time, the NIH was made of three buildings, one for each of the departments. Wilkins’s grant was in the Arthritis section (A-00180). The title was “Relation of Endocrine Glands to Growth and Development.”

In 1959, Dr. Wilkins suggested that I apply for a renewal (A-00180-9). It is of interest that my future work was supported by the same grant until I gave it up in 1995 as AM-00180-44.

Obviously, the emphasis of the research changed with time. At first, it was congenital adrenal hyperplasia. Later, it moved to androgens in the blood and to the first studies of “Testosterone Receptors in Complete Androgen Insensitivity.” We also were the first ones to have an assay for aldosterone in blood. The fellows were involved in all these studies.

## A triumphant visit to South America (November 1957)

Lawson was invited to visit South America. Lucile and Betsy were included. This was arranged by Dr. Salvador de Majo, the first fellow of the Pediatric Endocrine Clinic in 1948. The Wilkins family was welcomed like royalty everywhere starting in Buenos Aires. Cesar and Estela Bergada were hosts. There were many receptions and also several lectures. The trip went on to Cordoba, Argentina to visit José and Marina Cara. José had been a fellow in 1954.

After Argentina, there was Brazil, followed by Uruguay, Chile, and Peru. The visit to Peru was most vivid to Lawson. They went to Lima at the invitation of Dr. Nicanor Carmona. Lawson explained that Lima was a huge city of several million inhabitants. It was part of the Inca Empire. After the Spanish army’s victory, Pizarro founded the city in 1530. Lawson reported that the National University of San Marco was one of the oldest in the world.

Then from Lima, the Wilkins family went to Cuzco and Machu Picchu. Lawson had a great pleasure to describe the ascension by car, train, and cable car to the old Inca fortress at 2430 meters. The cable car ride was particularly breathtaking. He had to adjust to the high elevation and he had some trouble but managed to visit the Temple of the Sun, which was the birthplace of the Inca “Virgins of the Sun.” In the morning, Machu Picchu was in the clouds which dispersed with the sun. I obtained all the details as given by Dr. Wilkins, when he returned to the clinic.

## Gainesville, Florida (November 1958)

The fellows and staff of the Pediatric Endocrine Clinic had close contacts with the people of the Adult Endocrine Clinic. This was in part due to the fact that the pediatricians attended the weekly clinic of John Eager Howard in Hurd Hall, and the adult fellows attended the Saturday clinics of Lawson Wilkins. Two of the adult group were William Thomas and Tom Connor.

Eventually Bill Thomas left Hopkins for a position as the Chief Endocrinologist at the Medical School of Gainesville which had just opened. Bill asked me to come and give a lecture, which was fun. However, it also brought up the possibility that I could move to Florida. The school was brand new and attractive, and the dean was pleasant. I would become Associate Professor and would be given a lounge lab for which I would ask what were my specific requirements for research. This offer was attractive.

I suspect that Dr. William Thomas must have written to Lawson Wilkins about me. I was given a copy of the answer:

“Dear Bill:

As you well know, I cannot speak too highly of Claude Migeon from both his personal and scientific qualifications for the position which you are offering him. He has a thorough grasp of the fundamental problems of steroid chemistry and real scientific ability in investigating them. He shows great clarity of thought and his papers are examples of accurate, scientific writing. He has a good grasp of all the problems of endocrinology. His work has been a great aid diagnostically on both the pediatric and the adult endocrine side here. As you know, his own interests go deeper into basic problems of steroid metabolism, the binding of steroids in plasma and their transmission across the placenta.

If Claude should leave here, our work in pediatric endocrinology and our training of men in this field would be insuperably handicapped. I trust that every effort will be made to keep him here. I am expecting to retire from the administrative responsibilities of the pediatric endocrinological division next July, although I may have to continue responsibility under my present research grant until July 1960 and expected to continue my connections on the clinical side for another 5 years. I think, therefore, that the planning and offers for Claude Migeon will depend largely upon Dr. Cooke and whomever is to be selected for my position. I sincerely hope that you will not press Claude too rapidly for a decision but will give him an opportunity to learn what plans are being formulated here.

With my best personal regards,

Yours sincerely,

Lawson Wilkins”

Lawson asked me if I had read his letter and wanted to know what I needed to keep me happy. I suggested a promotion and increase in salary. My salary was very low and when I asked for a raise from Dr. Robert Cooke, he said that I was a bachelor with little needs.

However, when I realized that Lawson thought that he needed me, I said that I would stay at Hopkins, and I told so to Bill Thomas. Eventually my promotion came, and later I obtained an NIH grant of Clinical Research Scientists. Dr. Cooke also gave me a check of $10,000 to go study the transplacental passage of steroids during pregnancy in Stockholm at the Karolinksa Hospital with Dr. Carl Gemzell, chief of obstetrics.

## Betsy Wilkins’s wedding (December 1958)

Lawson was strongly in favor of marriage. He would bring up the idea of marriage very indirectly, reporting how much Lucile was a wonderful support for him. He would mention how he could always share the troubles of his life with her and would recall how much fun she was.

In early spring of 1958, I visited Seymour Lieberman at the Presbyterian Hospital in New York City. After the visit, I met with Betsy Wilkins who was working at New York Hospital. I felt that I should be kind to Betsy, not only because she was the daughter of Lawson but also because I was much older than she was.

Lawson told me that Betsy had met Philip McMaster in New York, a young man who had graduated from Hopkins and was an intern at New York Hospital. Later, he joined the NIH Department of Immunology. Phil’s father was a well-known scientist at Rockefeller Institute in New York City, who deeply impressed Lawson.

Lawson and Lucile were also impressed with their future son-in-law. As a romantic woman, Lucile was looking forward to a June wedding. However, Betsy and Philip decided to get married earlier.

The preparations took place actively and the wedding took place in December 1958. This was a great celebration. Lawson had his high hat, Lucile wore a magnificent dress, and Betsy had a splendid white wedding dress. Unfortunately, I had to get second hand information about the wedding and marriage, as I was in Chicago for the end of year celebrations with the Nanos family.

## Lucile’s death (June 10, 1959)

The first week in May 1959, Lucile had a seizure. It was not clear what this was related to. Dr. Wilkins, after a few days, took his wife to Johns Hopkins. He was very concerned and did not communicate much.

Because of the personal love of Dr. Judson Van Wyk for the Wilkins family, I wanted to give him the news. Here is my letter:

“Dear Jud,

I thought you would be interested to hear about the health of Mrs. Wilkins. Dr. Wilkins is so concerned about it that he has stopped coming to his office and does not take telephone calls and spends all his time with his wife.

As you probably know, the trouble started in Atlantic City when Mrs. Wilkins had a mild seizure which followed a short period of difficulty so far as sense of equilibrium and coordination. For this reason Dr. Wilkins did not go to the pediatric meetings at Buck Hill Falls and brought Mrs. Wilkins back to Baltimore to be hospitalized in Marburg where she was checked by a group of neurologists and neurosurgeons. At first it was thought that a subdural hematoma was at the origin of her trouble. An arteriogram, however, did not demonstrate any such hematoma. On Thursday, May 14^th^, a scanogram revealed the existence of a brain tumor which was thought to be located near very vital centers such as speech and coordination centers (I am not a great neurologists, I am just telling you what information I obtained from Dave Clark). That same day Dr. Wilkins and the neurologists had a meeting to decide on the possibility of an exploration. This was a most difficult situation for Dr. Wilkins. Eventually, he asked Dr. Park’s advice and it was decided to do a pneumoencephalogram followed by an exploration. These two procedures were carried out this morning. The neurosurgeon, Dr. Chambers, found a rather wild sort of tumor which he could not remove. A biopsy was performed and this is the present situation.

I don’t have to tell you how much Dr. Wilkins is upset. I wish there were something we could do to help him, but it seems that he would rather be left alone, at least at the present time. I will keep you informed of any further development.

As ever,

Claude”

Following my letter, a few weeks passed by during which Lucile had gone back home. Dr. Wilkins had passed the word for us not to interfere. Of course, we did not.

However, the symptoms were becoming more intense and the seizures more frequent.

I think a decision had been made to attempt an ablation of the tumor, in view of its present progression. So Mrs. Wilkins came back to the 3^rd^ floor of the Marburg Building and she was scheduled for surgery.

One evening, I was working late in the lab. I decided to go to the Marburg Building to visit Mrs. Wilkins at about 9pm. I managed to find her room. She was alone, awake. She was pleased to see me and to talk. She explained that it was strange; she was trying to pour tea without knowing whether or not she was pouring into a cup. We both decided that it was sad, but funny, and we laughed.

I tried not to get Lucile tired and I excused myself. Then she told me that her surgery was the next morning. I assured her that everything would be okay, even if I did not believe so. We said good-bye and I gave her a kiss. Then she said, “Claude, I am scared.” I patted her head and left, unable to speak as tears came to my eyes.

I believe she never recovered from surgery and on June 10, 1959, she passed away. The funeral was held at the church of the Redeemer. It was very sad and I cried.

## Barbara Ruben Migeon, MD and Dr. Lawson Wilkins (1959 – 1960)

Barbara had come to the House staff of the Department of Pediatrics at Hopkins in 1956. We dated for three years before a marriage decision was made. Between her interest in Pediatric Endocrinology and our dating, Barbara had contacts with Lawson Wilkins. For this reason, I have asked her to report her impressions and memories. So here are Barbara’s writings:

“I met Lawson Wilkins shortly after arriving at Hopkins as a Pediatric Intern in 1956. He had served as director of the Department of Pediatrics for the two previous years. Although he had already gleefully relinquished that job to Bob Cooke, he continued to give a Christmas party for faculty of the department, housestaff, as well as his endocrine fellows. It was a warm lovely occasion, with music and singing. I caught my first glimpse of Claude Migeon that night, admiring him as he sang “Chevaliers de la table rounde, dites moi si le vin est bon” with Victor Najjar at the piano. As a house officer, I remember Lawson’s making rounds with us, never bothered if there wasn’t a proper endocrine patient in the house. He would go up to the bed of some obese child and discuss why that child was not hypothyroid. As soon as I could, during my second year in the pediatric residency program, I elected a rotation in the endocrine clinic. I spent almost four months there.

Part of Lawson’s gift as a teacher was his ability to make his pupils feel smart. He managed to make me think that I, a young resident, could teach *him* something. Highly appreciative of any initiative, he made me feel very clever to have diagnosed a child with the hypertensive form of congenital adrenal hyperplasia, because I had accurately measured the infant’s blood pressure. I especially enjoyed his Saturday morning clinic held in the Harriet Lane Outpatient Department. I remember how he always narrated each patient’s entire clinical history, starting from the very beginning, so that any clinician coming in for the first time – even a novice – could understand the significant findings, and concluding with the present visit. Fortunately for me, one of his postdoctoral fellows was unable to carry out his responsibilities, and so I acted as a substitute fellow for several months. During that time, I learned from example how to meticulously care for patients and to learn from them at the same time. Because the experience was so satisfying, I proposed him the possibility of postponing my last year of residency and staying on as his fellow, at least until the end of the year. However, even though he needed help, Lawson discouraged that idea, insisting that I finish the residency program.

By the end of my Pediatric House-staff training, I was certain that I wanted to become an endocrinologist. I asked Lawson for a job, trying to convince him that it would be wise to take me as a postdoctoral fellow. But, he was equally certain that women should not be examining male genitalia. Having never had a female fellow, he encouraged me to get my training elsewhere. He seemed a bit uncomfortable telling me of his lack of enthusiasm for my being one of “the boys” (as he called his fellows). However, as he was not averse to having me in his clinic in another capacity, he suggested that I work in the lab with Claude. He was unaware at that time that Claude and I had an extramural relationship, which eliminated that possibility. As an alternative to staying at Hopkins, he suggested that I apply for a position with his former fellow, John Crigler, who was the chief of the division of endocrinology at Children’s Hospital in Boston. He also encouraged me to apply for an individual postdoctoral fellowship from NIH. Both applications were supported by generous letters of recommendation (he gave me copies of his letters). I received my fellowship and the position in Boston.

That might have been the end of our relationship, except for the fact that after only a year in Boston, I returned to Baltimore and Hopkins to continue my postdoctoral training in genetics with Barton Childs… and to marry Claude.”

## Marriage of Barbara and Claude Migeon with Dr. Wilkins (April 1960)

I dated Barbara for about three years. She was working with the Pediatric Endocrine Group. The fellows were William Cleveland and Robert Stempfel in 1959 to 1960. They both admired Barbara and advised me to marry her.

Barbara joined us at the Consultation, the Journal Club on Wednesday night when we went to dinner at the Chinese restaurant Me-Jon-Lo, or at the Steak House.

I guess that I was slow making the decision of marrying despite the advice of William and Bob. So that on July 1959, Barbara moved to Boston.

The separation brought back the decision to get married. Barbara was for eloping, as her family was concerned about our union.

So Barbara invited William Cleveland and his wife Marty to be our witnesses. Because a civil marriage could not take place in Baltimore at that time, we made arrangements with a Justice of the Peace in Alexandria, Virginia.

Barbara flew from Boston on a Thursday night. On Friday, we went to buy our rings. We made plans to drive to Alexandria with William and Marty Cleveland. However, there was an extra guest as described below by Barbara:

“Claude had always considered Lawson somewhat like a father, so he felt obliged to tell him that we were planning to elope. Lawson said, “Good, when do we go?” and he did come along for the trip to the Justice of the Peace in Arlington, Virginia. Along with Bill and Marty Cleveland we arrived one Saturday after sundown on April 2, 1960. The JP was an old man with Parkinson’s disease, and pinned to his lapel was the French Croix de Guerre. Our wedding vows were delayed for at least ten minutes while Lawson and his new friend discussed their adventures in France during the First World War. The wedding was followed by an elegant supper at Chez François, a well-known French restaurant in Washington D.C., where Lawson announced he was buying the drinks, and needless to say we had a merry time. Driving back to Baltimore very late that night, the five of us were in the midst of an animated discussion of several endocrine patients, when Lawson abruptly stopped the conversation, saying in his booming voice, “Well I’ll be damned! This is the first time I have discussed male pseudohermaphroditism with a bride at her wedding.” The following year, we were able to reciprocate by accompanying him to a courthouse in Washington where he married Teence Anderson.”

## Honeymoon in Scotland, Copenhagen, and Paris

“Not only was he a part of our tiny wedding party, but he went along on our honeymoon. As our excuse to spend several weeks in Europe, Claude and I were taking advantage of two endocrine meetings held back to back in July 1960. One was the meeting about the adrenal gland at the Royal Scottish Automobile Club in Glasgow, and the other, The First International Congress of Endocrinology in Copenhagen. For the weekend between the two meetings, we had made plans to visit Dr. James Farquar, a pediatric endocrinologist at his home in Edinburgh. Lawson rented a car, and we took a very scenic route, stopping at Loch Lomond in a fine “Scotch mist” – and on to Stirling Castle, where Dr. Wilkins regaled us with stories of the bloody deeds that had occurred there centuries before; he gleefully showed us the blood spots on the floor, which he accurately attributed to a murder committed there by King James the second. He fondly recalled having previously visited this bloody spot with Lucile. Always an astute observer, he pointed out the bales of hay in the Scottish fields, telling us that if he was parachuted into any country he would know where he was by the way the hay was packaged.

At the end of this delightful day, we arrived at the Farquars, and were warmly greeted by them. However, although it was the cocktail hour, no alcohol was offered, because as we soon realized, our lovely hostess Mary Farquar was a teetotaler. Lawson hinted that a drink might be welcome, telling our host, “In Maryland, we drink rye, but the scotch should be very good in Scotland” – however, none was forthcoming. At the end of the weekend, after lunch with the Farquars at the country home of the Regius Professor of Pediatrics, Richard Ellis, in the shadow of the ruins of Crookston Castle, (where Lord Darnley honeymooned with Mary Queen of Scots) we were driving through the beautiful Tweed River Valley. Stopping momentarily to enjoy the view, we returned to the car, but found that it was unwilling to move one step further – perhaps in rebellion because Lawson was not a gentle driver. When the mechanics arrived to take the car to a garage and us into Peebles, the nearest town, the car started for them without difficulty. While waiting for the chauffeur to take us back to Glasgow Airport, we decided to have dinner and a glass of that elusive scotch at the only hotel in town. However, it was seven o’clock on Sunday, and the kitchen was closed and because of blue-laws there was no drink to be had. Lawson and Claude headed for a nearby pub, where they charmed the pub keeper. Although impossible to get a glass of scotch, she suggested that, as travelers, they could buy the whole bottle. Meanwhile, I had managed to find some cheddar cheese and crackers. In the lobby of the hotel, we devoured the cheese and finished off the bottle by the time our car arrived. The chauffeur told us he hadn’t slept in three days, so Lawson kept up a continuous conversation to keep him awake during the journey to the airport.

In Copenhagen, Lawson saw patients as a consultant to Henning Andersen, the Danish pediatric endocrinologist. Henning considered himself a Wilkins fellow as he had spent several months at Hopkins. Enriched by his well-earned and unexpected consultation fee, Lawson invited us to join him and the Andersens for supper at Oscar Davison’s, a restaurant renowned for its lobster and *Aquavit*, which were both replenished whenever the plate or glass was emptied. The time spent together that summer was a memorable, intoxicating, and a bonding experience for us.”

Then, we went to France. Barbara was introduced to the Migeon family for the first time. She had problems with language as my family did not speak English. But she managed very well.

After five weeks away, we were ready to return to Baltimore. But before we had left, we had looked at houses with Lawson. One of them in Homeland was nice and we gave a bid slightly lower than the asking price. It was not accepted. At that point, we gave my family’s address in France as the contact address, if they wanted to change their minds.

And they did. So, in Rethel at my parents’ house, we found that we were the proud owners of a house. We had forgotten the details of the house and were anxious to face the facts and our new habitation.”

## Return to Baltimore (June 1960)

Our first move once in Baltimore was to go see our new house. We had forgotten some of the details of our home, after our honeymoon in Europe with Dr. Wilkins.

Barbara Migeon described our return as follows:

“In Baltimore, Lawson had been deeply involved in our search for a house, and in resodding the lawn of the one we purchased. To our surprise we awoke one morning to find that our front yard was piled high with several tons of dirt and sod, which he had ordered for us. The following Sunday, all day, Lawson, Claude, and John Eckert, an endocrine fellow, distributed the soil around the house, and sowed grass seed. Shortly thereafter, while Lawson was out of town, as a visiting lecturer, we received a telegram from him. It said, “Lovely weather for growing grass. Keep it wet.”

When we were in Copenhagen, we had bought several pieces of furniture made of teak wood at Illums Bolighus, a great department store. Lawson had insisted on paying for a lovely tea table. After he had seen our chimney, he decided that we needed to have: fireplace equipment. He bought all the necessary pieces, including a metal mantle that isolated the fireplace from the room, and he installed everything.

Because he was quite lonely, he came often for supper in our kitchen, particularly during weekends. He loved everything we cooked and appreciated a good wine with the meal. On September 21, 1960, we had dinner with the parents of Barbara. Her father, a family medicine doctor for many years, got along amazingly well with Lawson. They had the same feelings about the practice of medicine.

## Lawson Wilkins’s First Coronary (October 1960)

Following Lucile’s death, Lawson was rather despondent. So he spent a lot of time with us. He invited himself to our wedding, and our honeymoon in Glasgow, Edinburgh, the Tweed River Country in Scotland, the Loch Ness, and Copenhagen.

After, Barbara and I settled in our new house, Dr. Wilkins was often a guest. He attended the visit of the Bergada family from Buenos Aires, Argentina. He also was with us when the mother of one of his patients, the Marquisse di Fontiere from Lisbon, Portugal, visited our home with her son. There was also the visit of the Ambassador of Yugoslavia, Mr. Nikesic, as his sister Maria was a fellow in our clinic.

In addition, Lawson was often our “kitchen company” on Sunday nights, when we had a simple meal together. When he did not arrive on time one Sunday, we were concerned. Then, he phoned from Hopkins, in a private patient room at Marburg Building saying, “I won’t be there for supper, I had a coronary.” He told us not to worry and that he wouldn’t die. “I have met a wonderful woman, and I want to live. If I had not met that woman, I would have run up and down the steps of my house to finish my life, but now, I want to live.”

We thought that the old man was delirious, so we went to Hopkins and found him with an IV and an oxygen line and his pack of cigarettes on the nightstand. He repeated what he had said on the phone. I told him I was going to call Betsy and Phil in Paris. He insisted that if I told them, I would say they did not have to come home because it was a mild incident and that he was very well taken care of.

The next morning before work, I went to see him; he appeared to be in good shape. He told me to come back ten minutes before 5pm, because he needed me as a chaperone, when his wonderful lady friend would come to visit.

Of course, I came at the right time, just before a very nice, smiling lady arrived at his room. Lawson introduced me from his bed to Mrs. Catrina Anderson Francis. After some thirty minutes of simple conversation about health and how Lawson was doing, they talked about boats on the Chesapeake Bay. After a while, the nice lady excused herself and went away.

After she had left, Lawson wanted to know what I thought of her. It was clear that he expected me to say that Mrs. Anderson was wonderful. Lawson told me further that he was looking forward to leaving the hospital so he could propose to her. He asked if I would help him by chaperoning his meeting with “Teence,” her nickname. His joy was palpable, contagious, but was it unrealistic?

During his stay at Hopkins, John Eager Howard took care of him. Lawson did not like the hospital life, the nurses, the medications, or the requirement to rest in bed. Dr. Raphael David, his fellow, spent a great deal of time with Lawson. There were many visitors including Teence.

## Discharge home (November 1960)

Eventually, Wilkins was sent home to rest for a month. As Betsy was in Paris, Barbara and I moved in with him. It was very difficult to deal with Lawson who always wanted to do more than he was supposed to.

To make the situation more complicated, I had a meeting planned in France. I had to leave Barbara in charge with the help of a nurse. Here is a report from Barbara from that time:

“Dr. Wilkins asked us to chaperone Teence’s visits at the hospital and at home. As his daughter Betsy was living abroad at the time, and because we thought he might need companionship during his post-hospital recovery period, Claude and I moved in with him for the first two weeks after he returned to his home. I was there when John Eager Howard came to tell Lawson that he had won the prestigious Koch Medal from the American Endocrine Society. Dr. Howard asked that I leave the room, while he told him. I had no idea what the secret was, but I was sure that I would know all about it after Dr. Howard left. And I did, as Lawson was pleased by the news, and not good at keeping any kind of secret.”

During their close living together, Barbara had time to discuss many things with Lawson. This was the time when Barbara was becoming a geneticist. Here are her remarks:

“Quick to appreciate any novel treatment or other innovation that could facilitate the care of his patients or investigation of their disease, Wilkins was eager to have it for his patients. Shortly, after my return to Hopkins, I became one of those carrying out the analysis of human chromosomes soon after it was possible to do so, at first in the Cytogenetics Laboratory in the Moore clinic, which was directed by visiting professor Malcolm Ferguson Smith, and then in my own laboratory in the Department of Pediatrics. I recently reread one of the many notes I received from Lawson asking me to see that chromosome studies were carried out on a variety of his patients, which he suspected might have a chromosomal abnormality. Included were the females with Turner syndrome that he called “his synthetic brides,” proud that he could make them almost perfect females by treating them with estrogen. He had long before looked for the presence of a sex chromatin body, or Barr body in his patients, and had classified them as sex chromatin positive or sex chromatin negative. However at that time, the origin of the Barr body was not understood, so that chromosome analysis would be more informative. He was among the first to identify the nature of sex chromosomes in the true hermaphrodites and in his patients with non-classic Turner syndrome. As he suspected because they had a sex chromatin body, they were not 45,X but had 46 chromosomes with a second X chromosome, which most often was abnormal. And how excited he was to learn the results of these studies!

As you can see, despite his having deprived me of the pleasure of being one of his “boys,” Lawson had an influential role in my academic career, and we were very fond of one another. But early in our relationship, after a couple of drinks, he might say. “I love you and Ralph David (one of his “boys” who was an Egyptian Jew), but I don’t care much for Jewish people.” Once he told me that the German Jews in Baltimore, the department store merchants like the Hutzlers and Hoschilds, were fine people, but that the second wave of Jewish immigrants, the Russian Jews, destroyed his neighborhood (his family’s home was on Broadway). I answered, “but *I* am a Russian Jew,” and the subject never came up again. Clearly he felt uncomfortable about this bias, as he brought it up from time to time, almost apologetically. I have no doubt that it arose from his recollections of his childhood. However, it was only talk, as he never treated anyone with anything but respect, and one’s race or ethnic group never influenced the quality of care, or how he dealt with colleagues, students, and patients. Unlike the situation in other clinics, he did not distinguish between private and non-paying patients; they all got the same meticulous care and respectful treatment. I remember Lawson fondly as a gifted raconteur, extraordinary teacher, and outstanding clinical investigator. Especially memorable were his strong intellectual curiosity, his sense of history, his intense interest in all he surveyed, and his being my good friend.”

## Lawson and Teence’s wedding (April 1961)

Dr. Wilkins was a gregarious man who needed company at all times. When he lost Lucile in 1959, he was very lonely. He spent a lot of time with Barbara and me, including our wedding, honeymoon, his cardiac accident and recovery.

It also included his relationship with Teence and his marriage with her. Actually, we were pleased that he had found companionship with Teence, a divorcée of about 60 years of age. Her brother, Mr. Anderson, was a lawyer and friend of Lawson.

One day, Dr. Wilkins came into my small office on the 5^th^ floor of Harriet Lane Home. He settled himself on the only chair available and started to tell me about his decision of getting married again. It was going to be a civil marriage in Washington D.C. with the details supervised by Mr. Anderson. A court judge of D.C. would officiate.

Lawson asked that Barbara and I attend the ceremony. He gave me the time and place it would take place. We would all drive together from Baltimore. We were to leave several hours before the time, as we could not be late. He wanted me to drive slowly behind his car, so in case his car had a problem, we could drive them to Washington on time.

Thirty minutes before the hour agreed to meet, Dr. Wilkins arrived at our home on Thornhill Road. Barbara and I jumped into our car and the procession of Lawson first and we closely behind took off for Washington. We arrived safely, early enough to have time to meet with a few relatives of Teence, including her son of one of her two previous marriages.

The official ceremony was quite simple and short. At the end, we all embraced. Lawson was the happiest I had ever seen him.

Betsy could not attend the ceremony, because she was in Paris with Phil, who had a two-year fellowship in Immunochemistry at the Pasteur Institute. As for Barbara and me, we had to return to Baltimore promptly.

## The honeymoon of Lawson and Teence Wilkins (October 1961)

After the marriage in Washington D.C., Lawson and Teence came back to their Edgevale Road home. Barbara, Jacques, our one-month-old son, and I were invited one Sunday to their home. Also invited was a fellow, Dr. Paul Malvaux, his wife, and baby son. The Malvaux came from Leuven in Flanders in Belgium. Paul worked at the Université de Louvain. During lunch, the Wilkins talked about their plans for their honeymoon, asking us for advice.

Their first stop would be in Paris where they would meet with Betsy and Phil. Then there was the possibility of going to Provence in France and Rome in Italy. Lawson definitely wanted to go to Egypt to see the Pyramids and Sphinx in Cairo.

**Figure 8:**
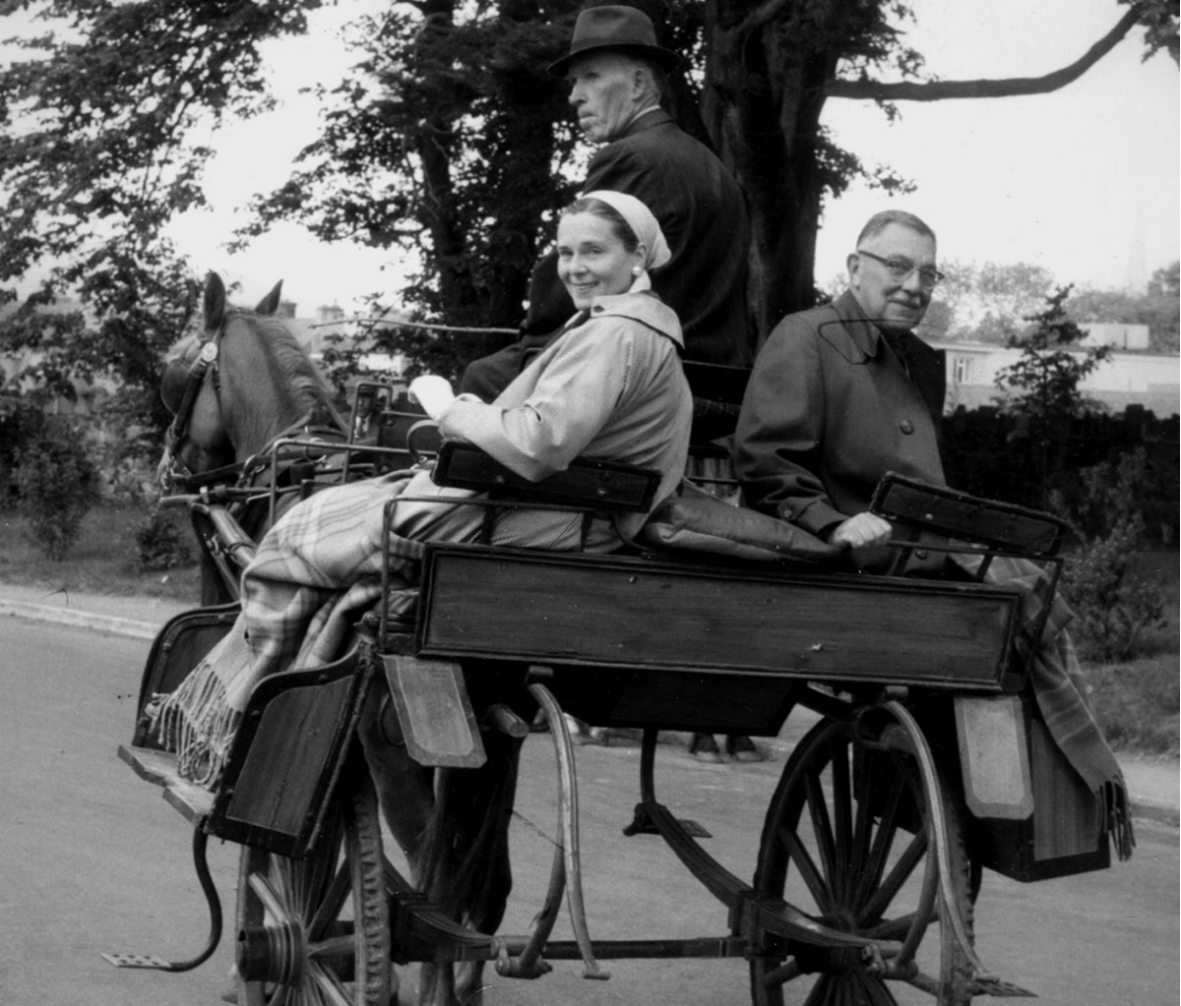
Lawson and Teence having a good time during their honeymoon voyage in Ireland. (1961)

After lunch, we wished them a good trip and left. I heard about the trip after their return. Lawson was happy to see Betsy and Phil in Paris. They stayed several days and had a great time exploring the city with them. They also went to visit Ireland. (Figure 8)

Next, I heard a lot about the Pyramids and the Sphinx with its broken nose from a cannon shot from Napoleon I. Teence had ridden on the back of a camel, while Lawson refused to do so, thinking it was not dignified for him. I told Dr. Wilkins that this reminded me of an experience I had had when we went to a very small island, Hydra, in the Cyclades, south of Athens. This island had no cars. When Barbara and I disembarked from our boat, the hotel had sent several donkeys to pick us up. First, the man put our luggage on the first donkey. Then he picked up Barbara and put her on the back of the second donkey before she could say anything. When he came to grab me, I refused very loudly, and the man did not resist. We started our walk towards the hotel. When one of the donkeys had droppings, the caravan stopped, the man collected the stuff very carefully and deposited it in bags which were on the back of a fourth donkey. At the end of my story, Dr. Wilkins had a good laugh and he understood that I did not think that it was dignified to ride a donkey either. At the same time, Lawson reminded me that Jesus had ridden a donkey in the Bible.

## Death of Lawson (September 27, 1963)

Early in the morning of September 27, I got a phone call from Teence Wilkins. She asked me if I could come to Edgevale Road because Lawson felt much worse and could not move from bed. I said I would come immediately. I drove there and saw another car already parked in front of the house. I ran up the steps from the street to the house and rang the doorbell. Teence opened the door and told me that Dr. John Eager Howard was there and she took me upstairs to Lawson’s bedroom.

There, I found John Eager Howard auscultating Lawson who had great difficulty breathing. My “good morning Dr. Wilkins” did not get a response. When John Eager Howard was finished, he turned to me and gave me a syringe and a couple of test tubes. He requested that I draw Lawson’s blood and carry it to the lab at Hopkins.

John Eager Howard and I were very concerned to see the chief in such a low status. Dr. Wilkins could not respond to me. I had to get his arm, put it in position, and I took the blood without any trouble.

I told Dr. Wilkins that I hoped he was going to feel better soon. Then I left for Hopkins Hospital and delivered the blood with the prescription of John Eager Howard. Afterwards, I went to the third floor of the Children’s Medical and Surgical Center and told Bob Blizzard and my other colleagues, as well as the secretaries and technicians that Dr. Wilkins was gravely ill. We were all greatly concerned.

A few hours later, I got another phone call from Teence. She told me that Lawson had been transported to Hopkins to be hospitalized. I went to Osler 4 rapidly. I met with Dr. Richard Ross, the cardiologist, in the corridor and asked about Lawson. His statement was very terse: “Dr. Wilkins is in complete cardiac failure.”

I went to his room. It was a sad spectacle. The chief was as pale as his sheets, lying down with oxygen therapy and a gastric tube. He was unable to speak. He saw me and made a motion asking for a piece of paper and pencil. After I asked how he felt, he wrote, “Claude, I am very tired.” I tried to say something appropriate, but I admit that I did not know what to say. I ended up saying, “You are going to feel better, Dr. Wilkins.” He looked at me, shook his head and closed his eyes, clearly in pain. At that point, the nurse ushered me out of the room. That was the last I saw of Lawson Wilkins.

I went home to Barbara and my two little boys, Jacques and Jean-Paul. I could have cried at the sight of the contrast between near-death at the hospital and new life in our home.

The next morning, Teence called me and reported that Lawson had passed away peacefully in the night. Betsy McMaster had seen her dad before he died. Teence asked me to let the past fellows know as soon as possible. I went to Hopkins and personally called Alfred Bongiovanni, Jud Van Wyk, George Clayton, Melvin Grumbach, and William Cleveland. The secretaries had a list of numbers and made calls to many fellows and colleagues of Lawson as well.

It was very difficult for me to focus on what to do. I went to the Wilkins’ home, where there was a lot going on and I felt like an outsider. I returned to Hopkins for a while and then went home where I had to help with our two year old Jacques and two week old Jean-Paul.

Eventually, Teence and Betsy let me know that the funeral would be at the Church of the Redeemer on Charles Street. Barbara could not attend because of the new baby. I went by myself and met a number of the past fellows.

During the service, I could not stop tears from rolling down my face. I tried concentrating on the stained glass windows on the right side of the chapel, but this did little to prevent the tears.

Teence arranged to have the past fellows who were present to carry the casket of the chief. I tried to hide my sadness, unsuccessfully. We were directed toward a car to carry the casket to the cemetery. And this was the end.

On October 5, 1963, I received a kind note from Betsy McMaster:

“Dear Claude,

I cannot begin to tell you how much it has meant to all of us to have your help and companionship throughout the past few days. You had such a special place among “Daddy’s boys” and we appreciate beyond all words all you have done for him over the years.

Sincerely,

Betsy”

It is quite ironic that our son Jean-Paul was born on September 12, 1963 and Lawson had visited mother and child in their room at Hopkins. Dr. Wilkins died two weeks later, September 27. Five weeks later, his grandson Charles was born.

Eventually, I went back to work at the Pediatric Endocrine Clinic, which had just moved from Harriet Lane to the 3^rd^ floor of the Children Medical and Surgical Center (CMSC-3). I joined Bob Blizzard, the fellows, and the technicians, keeping memories of Dr. Lawson Wilkins.

(Continued in Part 3, http://www.ijpeonline.com/content/2014/S1/S4), [1])

## Competing interests statement

The author has no competing interests to disclose.

## Declarations

This article has been published as part of *International Journal of Pediatric Endocrinology* Volume 2014 Supplement 1, 2014: Remembering Doctor Lawson Wilkins: a pioneer of pediatric endocrinology. The full contents of the supplement are available online at http://www.ijpeonline.com/supplements/2014/S1.
